# Weathering the storm: A qualitative study of social prescribing in urban and rural Scotland during the COVID-19 pandemic

**DOI:** 10.1177/20503121211029187

**Published:** 2021-06-30

**Authors:** Alison Fixsen, Simon Barrett, Michal Shimonovich

**Affiliations:** 1 College of Liberal Arts and Sciences, University of Westminster, London, UK; 2 Newcastle University, Population Health Sciences Institute, Newcastle Upon Tyne, Tyne and Wear, UK; 3 MRC/CSO Social and Public Health Sciences Unit, University of Glasgow, Glasgow, UK

**Keywords:** Social prescribing, qualitative studies, COVID-19, community services

## Abstract

**Objectives:**

The non-clinical approach known as social prescribing aims to tackle multi-morbidity, reduce general practitioner (GP) workload and promote wellbeing by directing patients to community services. Usual in-person modes of delivery of social prescribing have been virtually impossible under social distancing rules. This study qualitatively examined and compared the responses of three social prescribing schemes in Scotland to the COVID-19 pandemic.

**Methods:**

We interviewed a theoretical sample of 23 stakeholders in urban and rural social prescribing schemes at the start of COVID-19 pandemic. Follow-up interviews with a representative sample were conducted around 10 months later. Interviewees included social prescribing coordinators (SPCs) GPs, managers, researchers and representatives of third sector organizations. Interview transcripts were analysed in stages and an inductive approach to coding was supported by NVivo.

**Results:**

Findings revealed a complex social prescribing landscape in Scotland with schemes funded, structured and delivering services in diverse ways. Across all schemes, working effectively during the pandemic and shifting to online delivery had been challenging and demanding; however, their priorities in response to the pandemic had differed. With GP time and services stretched to limits, GP practice-attached ‘Link Workers’ had taken on counselling and advocacy roles, sometimes for serious mental health cases. Community-based SPCs had mostly assumed a health education role, and those on the Western Isles of Scotland a digital support role. In both rural or urban areas, combatting loneliness and isolation – especially given social distancing – remained a pivotal aspect of the SPC role.

**Conclusion:**

This study highlights significant challenges and shifts in focus in social prescribing in response to the pandemic. The use of multiple digital technologies has assumed a central role in social prescribing, and this situation seems likely to remain. With statutory and non-statutory services stretched to their limits, there is a danger of SPCs assuming new tasks without adequate training or support.

## Introduction

The non-clinical methodology known as social prescribing presents a number of advantages in these challenging times. Widely endorsed by government and health policy makers, the aims of social prescribing include tackling multi-morbidities,^1^ reducing GP workloads,^2^ mitigating health inequalities^3^ and delivering patient-centred care.^4^ However, the typical modes of delivery of social prescribing – such as person to person interviews and live activities and classes – have been virtually impossible under social distancing rules, while evidence has emerged concerning exacerbation of existing social and health inequalities by the pandemic, presenting additional challenges within the already tough landscapes in which social prescribing schemes in Scotland operate. We considered it important to understand the conditions under which social prescribing in urban and rural Scotland had been operating during COVID-19 and how resultant changes might inform the future of social prescribing.

This article explores the experiences of professional stakeholders of social prescribing in both urban and rural areas of Scotland during the first year of the pandemic. It highlights the complexities and inconsistencies of social prescribing and the challenges the pandemic has presented to different schemes, including remote delivery and home working, and taking up additional responsibilities where statutory and non-statutory services are stretched to their limits. Two key questions are explored: how have the conditions created by the pandemic reshaped and repurposed social prescribing and how have they impacted on the role and responsibilities of the social prescribing coordinators (SPCs) in urban and rural schemes?

### Background

Evidence continues to emerge concerning the direct and indirect health and community effects of COVID-19, and the significant burden it has placed on vulnerable groups including those with pre-existing health conditions.^5–8^ This evidence highlights race and ethnicity as significant factors in COVID-19-associated morbidity^9^ and the widening gap between different communities and social groups in the United Kingdom.^10–12^ With its aim of addressing individual health and wellbeing issues related to social, economic and psychological circumstances,^13^ social prescribing presents a number of advantages in the wake of the pandemic. By social prescribing, we refer to an approach that allows healthcare and other professionals to refer patients towards health and wellbeing interventions and activities in their local community. Usually, a designated SPC^14^ or ‘link worker’^15^ works with the client to agree a personalized social prescription and facilitates (where available) access to community resources including sport and leisure, welfare, education, culture, employment, green gyms, allotments and so on, while some participants may also be referred back to mainstream medical or mental health services.^16^

Within the United Kingdom, there are multiple social prescribing schemes, following different models. The most common is the GP practice-attached model, where a ‘Link Worker’ is located in a particular practice. Other social prescribing schemes, usually funded through the private and charity sectors, support a cluster of GP practices and/or cater for people with specific health, welfare or age-related issues.^17–20^ Typically, GPs, pharmacists, social workers and other professionals can refer ‘suitable’ candidates into schemes. To further complicate things, what for convenience we refer to as SPCs are, in practice, known by a range of titles such as link worker, social prescribing advisor, community navigator and community connector.^21,22^

Critics of existing social prescribing point to its reliance on community goodwill,^22^ and use of individual-level health interventions to address socially and politically embedded issues that, in reality, demand the implementation of government-level interventions.^11,22^ Nevertheless, there is plenty of evidence supporting the health and social benefits of social prescribing to individuals and groups in particular communities,^7,22,23^ while its role in extending the boundaries of traditional general practice and strengthening community-professional partnerships has been substantiated in various studies.^5,17^ This is significant, as traditionally the links between primary health care and the community and voluntary sector have been underdeveloped.^24^

One under-explored area of social prescribing is its use of digital technology, which is surprising as e-prescribing has been the topic of studies for some years.^25,26^ E-prescribing may also seem at odds with the interpersonal model of health and wellbeing on which social prescribing is based, where emphasis is placed on face-to-face contact and access to physical groups and spaces.^4,27^ Necessitated by COVID-19, interactive technology (IT) has become virtually indispensable^28^ with the crisis radically changing the way in which both health services and research tools have been delivered and applied.^6,28–30^ The ubiquity of online delivery has multiple implications, including creating greater social and health divisions between the digitally privileged and disadvantaged. With this in mind, initiatives such as *Connecting Scotland* have been set up to distribute iPads to the most vulnerable through institutions and organizations such as GP surgeries, care homes and charities^31^ while GP surgeries and social prescribing schemes have been busy training their staff in IT skills and data protection measures.^32^

#### Social prescribing in Scotland

Recent data from Scotland suggest that health inequalities have only marginally reduced there in the last decade.^33^ Prior to the pandemic, National Records of Scotland (NRS) figures showed life expectancy to vary by seven years depending on the council area in which a person is born in – and by 10.5 years depending on how deprived the area.^33^ COVID-19 has further highlighted and exacerbated inequalities that exist within the country.^28^ The term ‘Deep-End’ practice refers to the concentration of poverty and other markers of deprivation within a practice area. In Scotland, these practices have around 88%–44% of their patients in the most deprived 15% of data zones.^34,35^ The Glasgow Links Worker programme was first rolled out as a pilot across seven Deep End practices in 2013 and has since been the object of intensive evaluation.^3^ Despite some mixed findings and conclusions from these studies,^3,17,36^ under the new GP contract, the Scottish government pursued its commitment to deliver 250 link workers over the life of its Parliament to GP practices or clusters of practices across Scotland. Around 30% of Deep End practices now have a Community Links Practitioner (CLP) or ‘links worker’, whose role is to support the navigation of patients with needs beyond medicine to appropriate local agencies and community organizations and enterprises. In addition, social prescribing is delivered through multiple third sector schemes running out of urban areas, including as Glasgow, such as the SPRING social prescribing programme described later in this paper.

Health inequality issues affecting rural and isolated populations in Scotland are different to deprived areas of cities, but are equally significant. Among the Scottish islanders, 25% of people are aged between 45 and 59 years, while in the Outer Hebrides, those in the 65–74 years category represent around 13.7% of the population.^37^ Depopulation and an aging society have led to an increase in those living alone and with chronic health conditions, along with a shrinking health and social services workforce and a declining number of unpaid family carers to support them. Recent census data suggest that the Western Isles has the greatest proportion of lone pensioner households in Scotland which, even without social isolation or shielding, presents a challenging situation.^19^ Living alone has strong associations with social isolation and loneliness, higher risk of dementia, depression and premature mortality. Fuel poverty on the Isles is another significant problem, contributed to by high domestic fuel costs, generally low-household incomes, older properties and damp maritime weather conditions.^38^

## Methods

We used a qualitative study design to explore barriers and enabling factors affecting the delivery and access of social prescribing services to those living in urban and rural areas of Scotland during the pandemic. We captured responses within the three social prescribing schemes described in Table 1: The Links Worker Programme, SPRING social prescribing and Community Navigator programme, with the idea that their lessons from the pandemic could inform how social prescribing might be reimagined as we emerge from it. SPRING is a third sector social prescribing scheme operating in various community settings in urban areas and funded by the National Lottery. The Community Navigator scheme managed through mPower – a 5-year project supported by the European Union^37^ serves rural communities including on the Western Isles (see Table 1 for full details). Going forward, we will refer to SPCs associated with the Community Links Worker Programme as CLPs or ‘links workers’, those associated with SPRING as SP advisors and those associated with mPower as CNs (Community Navigators). When discussing the role in general or across the three schemes, they will be referred to as SPCs (Social Prescribing Coordinators).

**Table 1.** Summary of three social prescribing schemes across Scotland: Community Links Worker Programme, SPRING and mPower.

	Links Worker Programme	SPRING	mPower
Description	Started as a pilot in 2014 as a way to support GPs in practices in Glasgow’s most deprived areas. Currently, 34 CLPs/link workers are attached to practices in Glasgow	SCHW in Scotland and HCLA in Northern Ireland teamed up to fund social prescribing schemes in 30 rural and urban areas, including Glasgow	The mPower project supports social prescribing and eHealth interventions across borders in the West of Scotland
Scheme location for this study	Glasgow Deep End general practice surgeries	Community centres in Glasgow	Isles of Barra, N. and S. Ulst, Benbecula, Harris and Lewis
Estimated scheme duration	October 2014 to December 2021	April 2018 to April 2023	Late 2017 to December 2021
Funders	Scottish Government	The National Lottery Fund	European Union’s INTERREG VA Programme, managed by the Special EU Programmes Body (SEUPB). Match funding is provided by Scottish Government
Management organizations	Health and Social Care Alliance	Scottish Communities for Health and Wellbeing (SCHW) in Scotland	NHS Western Isles employs the social prescribers. The University of the Highlands and Islands is evaluating the project.
Financial investment	The Scottish Government paid each participating practice between around £50,000. Up to £17,500 is spent on adapting the Links Worker Programme into the surgery	The National Lottery Fund provides £3 million in support	The project budget is €8,700,000 (for all three regions)
Social prescriber location	Based in general practice surgeries	Based at voluntary and community organizations	Serve rural communities and are based in regions
Designated name of SPC	Community Links Practitioner or links worker	Social Prescribing Advisor	Community Navigator
Patient criteria	All patients over 18 years registered with the general practice, including those with complex needs. Patients can be referred or self-refer	Adults over 18 years who have been referred. Focus on patients with low-level needs, e.g. social isolation, loneliness and physical activity	Adults over 18 years, but predominantly over 65s with long-term conditions

SPC: social prescribing coordinator; NHS: National Health Service; HCLA: Healthy Living Centre Alliance; SCHW: Scottish Communities for Health and Wellbeing; INTERREG: European Territorial Cooperation.

### Ethics

All parts of the study were approved by the University of Westminster Liberal Arts and Sciences Ethics Committee. All participants were supplied with participant information sheets and gave their consent to the interviews being recorded and for extracts of interview data to be used. All data use adheres strictly to the terms of the Data Protection Act (DPA 2018). Only pseudonyms are used in this study.

### Data collection

A theoretical sampling approach (with a mixture of purposive and snowball sampling) was used to recruit participants who had different interests and involvements in social prescribing first in the Glasgow area and later the Western Isles of Scotland. A loosely structured interview guide (see Supplementary File 1) was designed for the interviews. This was based on a similar interview guide used by the first author in a previous study but was adapted to the conditions and background of participants. The guide was approved by the University ethics committee. All 23 participants were first contacted by email by the first author, who explained the researchers’ backgrounds and interests in the study and attached a Participant Information and Consent Form. Due to social distancing conditions, all interviews were conducted individually by the first author (she is a senior lecturer and experienced qualitative researcher) via Skype or telephone. All those contacted agreed to be interviewed. Data gathering commenced in late March 2020 and was completed early March 2021.

Interviewees included SPCs, GPs, social prescribing managers, researchers and representatives of third sector organizations. Twenty-two interviews were conducted near the start of the pandemic, with seven follow-up and one new interview conducted in 2021 with representatives from each scheme to gain a more longitudinal perspective of how different schemes and their service users had fared during the pandemic (see Table 2, participants). Interviews were 30–60 min in duration and were audio-recorded. Collection of data was carried out until saturation had been reached. Participants were given the opportunity to review their transcript if they chose.

**Table 2.** Table of participants with dates of interviews and role description. All names are pseudonyms.

Pseudonym	Interview date	Role
Jane	01/04/2020 18/02/2021	Manager
Martin	05/04/2020	GP
Anna	07/04/2020 02/02/2021	Community Links Practitioner (CLP)/links worker
Steph	09/04/2020	Community Links Practitioner (CLP)/links worker
Liz	20/04/2020 12/02/2020	Social prescribing advisor, SPRING
Maria	21/04/2020 03/02/2021	GP
Lynne	28/04/2020 27/01/2021	Manager
Harriet	28/04/2020	Manager
Paula	28/04/2020	Researcher
Brenda	01/05/2020	GP
Mia	05/05/2020	GP
Nina	07/05/2020	Community volunteer
Susan	11/05/2020	GP
Ray	14/05/2020 04/02/2021	Community Links Practitioner (CLP)/ links worker
Jade	29/05/2020	Community volunteer
Nick	19/05/2020	Researcher
Jerry	24/05/2020	Manager
Clare	26/05/2020	Manager
Isabelle	04/06/2020	Community Navigator (CN)
Fiona	11/06/2020 05/02/2021	Community Navigator (CN)
Viola	16/06/2020	Community Navigator (CN)
Alison	18/06/2020	Researcher
Ranesh	04/03/21	Community Links Practitioner (CLP)/links worker

GP: general practitioner.

### Data analysis

An inductive thematic approach^39,40^ was used, where themes were derived from the data, and emerging themes discussed and agreed among the research team. Analysis was conducted in two stages (1) ordering the data set to categorize it into themes and (2) ‘making sense’ of the data by a close reading of each theme and the drawing out of interpretations.^37^ This involved each member of the team reading and re-reading transcripts to familiarize ourselves with the data and making notes of recurrent themes within and across participants’ transcripts.^38^ To handle such a breadth of data, and to ensure a systematic approach to the analysis, each transcript was uploaded to NVivo 12 by the second author. A high-level approach was adopted at this stage, which involved coding each transcript into broad themes/domain summaries.^40^ For example, the domain summary COVID-19 and social prescribing was organized into the themes: challenges experienced due to COVID-19; immediate impacts and responses to COVID-19; impact of COVID-19 on social prescribing workers; opportunities presented by COVID-19 and uncertainties for the future due to COVID-19.

The next stage involved mapping out the data from each participant that corresponded with each theme, by creating a framework matrix (see Supplementary File 2, for an example). This process allowed for identification of data gaps, distribution of data across the sample, similarities and differences in participants’ responses and deviant cases. As further themes emerged from each transcript, new codes were created accordingly, in an iterative process. This refinement stage of the analysis corresponds to ‘creating order’ as described by Spencer et al. 2013.^39^ In this way, themes were generated, meaning was assigned to themes, and the data which portrays this meaning was coded to each theme. It was then possible to start to develop explanations from the data and explore its wider implications.

## Results

### Complex social prescribing landscape

Our findings revealed a complex social prescribing landscape in Scotland with various schemes funded, structured and delivering services in diverse ways (see Table 1 for some comparisons). Even to those with direct experience of social prescribing over many years, the structures and funding arrangements around this modality were perceived as multifarious. Participants described consortiums of organizations, different funding cycles, streams and partners, and complicated contracts and sub-contracts where they worked in one organization but were funded by another, for example, ‘So it does sound convoluted, because it is. It’s taken me some time to get my head around it as well’ (*Liz, SPRING*). These funding and operational complexities were not exclusive to those working in third sector organizations; link workers working within a single GP practice described complexities of how their role is viewed, funded and operationalized, as well as variations in the ways they were treated by staff and the expectations placed upon them. This rather loose arrangement was perceived by this link worker as initially confusing:

You quickly found out as well that lots of different links practitioners’ roles – [although] their role is the same in terms of what you do – how you carry out that role could be different depending on what practice you’re in, the staff that’s within that practice and their knowledge of how much you can help a patient and where the referrals come from. (Steph, Link Worker)

We particularly noted different and strong opinions concerning medical versus community models of care, and the advantages and disadvantages of having link workers attached to a particular GP practice were a frequently discussed topic. To those with investment in practice-attached schemes, having the link worker ‘embedded within the practice’ with ‘open lines of communication with the GPs in the practice’ was seen as of high importance. Not only did this mean less delay for the patient between GP referral and meeting with the links worker, but also it helped to create a sense of trust for all concerned:

Other charities, other third sector groups do their own version of social prescribing, which anyone can refer into . . . The idea [with Deep End] was having a Links Worker role who is practice-attached [to the GP surgery], who became a trusted member of the practice team, so you could very much say [to a patient], ‘Listen, I know the person that can help you, their name is x, they’re just down the corner here, I’ll come and introduce you to them now’. (Martin, GP)

### Variations in referral criteria

GPs working in Deep End practices emphasized the complex caseload they routinely worked with, which included a large number of traumatized patients and people who rarely left their homes, many of whom were conditioned into thinking that medications were needed to fix their problems. To free up such GPs, a decision had been made early on that link workers should see any client who came to them, making their client base diverse:

Any age group around any condition, any social issue, anything that is having an impact on their health can, we’re not time limited so . . . supporting them to link in with different organizations or being that person that they can come and speak to and offer that kind of emotional support in periods of crisis. (Ray, CLP/links worker)

Those employed through SPRING had more defined referral criteria and their work largely focused on clients with ‘low level mental health and social issues’ – although in practice they were sent a good number of patients with complex problems. One advantage of the SPRING model was that their social prescribing advisors served a number of GP practices within a local area. Locating the advisors within community settings was also seen as an excellent means of asset-linking:

When someone is referred to SPRING, the social prescribers can instantly refer them to activity services, support services within their own organization or out with. So that’s a real positive because the link workers really need us to refer their people into them [the community activities] as well, because they need those services and initiatives that are there. (Jane, SPRING manager)

Accounts suggested that Deep End link workers were also far from desk-based; in addition to seeing people in the GP practice they visited the client’s home, met in a café or library, arranged walk and talk sessions and personally accompanied people to community activities. These accounts illustrate how, although in essence SPCs across the schemes all engaged in similar work – which is linking service users to people and organizations that can support them – there were conflicting views among stakeholders on how this can be most effectively achieved.

### Background and training

Another area of difference concerned background and training. While all SPCs had some basic knowledge of health behaviour change, links workers and SPRING advisors came from community rather than medical backgrounds, whereas the CNs interviewed were trained nurses or had worked in National Health Service (NHS) settings. This, and having a cohort with at least one long-term condition, might be why, of all the three schemes, the CNs alone showed a familiarity with medical testing and medical digital apps:

There’s a really useful site called *My Diabetes My Way* . . . [or] someone maybe had high blood pressure then we could lend them a blood pressure monitor, link them in on *Florence*, which is a text messaging service, so they could take the readings, feed the results in . . . Or we could suggest that based on the findings and it’s very much based against defined parameters, that they might want to go to the GP. (Clare, mPower manager)

### Points of concordance

Despite these differences, there were important similarities among SPCs. There was universal agreement about the importance of having a person-centred approach to social prescribing. Due to their long consultation slots (usually 40–60 min) and complex service-user base, SPCs could deal with social issues and problems at more length and with greater effect than where the person simply kept returning to their GP. The ability to get to know service users and their needs, read body language and non-verbal clues, and sometimes physically assist them or accompany them to sessions, was seen by all SPCs as necessary for effective support:

Our social prescribers, so it’s not about sign posting people. . . . the social prescriber will sit down with a patient and they’ll sit and chat about, right, what is important to you, what is it you want to do to improve your health and well-being. (Jane, SPRING manager)

In order to signpost service users to be the best resources, all SPCs required a good knowledge of the local landscape. The challenge was then recruiting people with this background, or as manager Jerry explained, aiding their transition into a community-connected role:

The Community Navigators . . . some of them have come from the Health Service . . . some of them do know their communities very well but some don’t. They come from an institutionalized, if you like, background working for the NHS and, so somebody like me who understands how the voluntary sector works . . . we kind of advise them [the CNs] on how to approach this whole business. (Jerry, mPower)

There was also a general agreement concerning the importance of buy-in from local GPs, so that (1) GPs would refer on patients and (2) GPs would understand the importance of social prescribing. Prior to initiating the Links Worker Programme, a group of GPs had set up the Deep End scheme in order to tackle the, Inverse Care Law, which states that good medical care tends to vary inversely with need in the population served According to GP Martin, the rollout of the Links Worker Programme had been ‘probably the biggest success’ of the Deep End initative, as it had since been rolled out nationally; medical students had even been shadowing links workers in Deep End practices. Even so Martin was aware that buy-in to social prescribing from GPs generally remained patchy: ‘I think we probably been operating on a coalition of the willing’. This is not to suggest that the individual GPs we spoke to expressed reservations about social prescribing, they were all enthusiastic; however, several stakeholders spoke about GPs who remained ‘very medically minded’.

#### Effects of the pandemic

For both GP and SPCs, working effectively during the pandemic had been challenging and demanding. This was especially the case for those based in areas of high deprivation and/or multi-ethnicity, where everything had been ‘more of a battle’. In particular, speaking on the phone with patients/clients who were less fluent in English could be slow and unsatisfactory work:

It’s very, very intense. It’s challenging and a completely different way of working, and especially challenging for our patients because we either have language barriers, so that means we need interpreter consultations and it was basically the cues from the patients, from facial expressions in interpreter consultations, so that is very, very challenging. (Maria, GP Deep End)

From the start of the pandemic, schemes had been busy supporting existing and shielding clients through phone calls, food parcels, online classes and more. The initial chaos of lockdown had led to a drop in numbers of referrals into the link worker scheme, which manager Lynne believed had mirrored changes in GP practices; fewer appointments than usual and more phone consultations meant that GPs were less able to pick up social cues. GP Mia spoke of the difficulties of using a psychosocial model in these fraught times, admitting that she had fallen back on a more medical model due to high workload, time constraints and the absence of face-to-face communication: ‘I am quite sure that my prescriptions for antidepressants has gone up’. Having access to the services of a link worker freed GPs up to focus on more acute medical needs arising from COVID-19:

He [the links worker] has been invaluable during this Covid pandemic, even though he needs to work from home. But despite that he’s been amazing at liaising with third sector organizations, and patients, and mobilizing resources to mitigate some of the effects of the pandemic around practical things like foods deliveries, prescriptions – but also emotional support . . . mental support. (Mia, GP)

### Remote working

One of the most significant changes in social prescribing brought about by COVID-19 has been the loss of face-to-face contact between practitioners and clients. In GP surgeries where a link worker was in place, the scheme had ‘worked well [but] we are missing the direct contact because our link worker works remotely’. According to Ranesh, communication had been ‘a lot more impersonal because it’s everything over texting through the GP service system’. Where possible, SPCs had sought out interactive ways to connect with their clients; however, at the start of this study, there were wide differences between schemes in terms of their digital capacity. Before COVID-19, mPower had already been distributing tablets to clients in remote areas, and by the end of the study, all schemes were handing out iPad devices through the *Connect Scotland* service. Link workers and CNs had been making use of the NHS video platform *Attend Anywhere*, while the Annexe had its own digital platforms. However, video calls presented their own challenges with technology sometimes failing and home working less of a professional and private space than an office.

### Changing demands

Even before COVID-19, the impacts of closure of local statutory and non-statutory community services were highlighted in studies including our own^11,22^ and the general view of participants was that the situation in Scotland had become far worse. The pandemic had seen mental health services stretched to their limit with waiting times of up to a year in Glasgow. By January 2021, it was noticeable that people were ‘really struggling’ with such things as suicidal ideation, a sense of hopelessness and heightened levels of anxiety becoming daily referrals:

[Now] the referrals coming through are . . . more round mental health – I know that was prominent right at start but it’s kind around social isolation; the groups are not face-to-face, and a lot of people are digitally excluded for one reason or another. So a lot of high distress calls – people fleeing violence, relationships breaking down. (Anna, CLP/links worker)

Other areas of referral were related to food poverty and unemployment; Ranesh, a link worker based in a multiethnic area, had seen more referrals to food banks and ‘different community organizations that provide food for free for people’. Giving money advice and benefit advice had become a greater part of the work: ‘Since Covid it [financial stress] has very much increased’.

The role of SPRING social prescribing advisors had also altered during the pandemic, but in different ways to link workers. Whereas by January 2021, the number of GP referrals to link workers had risen, SPRING advisors had experienced a steep drop in GP referrals, but had continued to serve shielding clients through the *PATCH* programme and, as well as supporting vulnerable people with food and gift parcels, had assumed a new online health education role though SPRING’s *Connect Well* workshops:

We did some online activity as well . . . cooking and nutrition classes and . . . Exercise, so yes, and . . . around the summer last year we done some afternoon tea, which was just a selection of sandwiches and cakes and things to offer to the most vulnerable . . . and they really enjoyed that, it made them feel a little bit special as well. And at Christmas time we done some pamper packs . . . (Liz, SPRING)

To a limited extent, SPCs had been able to arrange live meetings with their clients. Both link workers and SPRING advisors had delivered walk and talk sessions for the most vulnerable and outside of full lockdown, and link worker Ray had organized a socially distanced walking group. In most (but not all) areas of the Western Isles, CNs had been ‘very fortunate that we do still continue with face-to-face contact’, so initial meetings were in person and follow-ups face-to-face. During the winter months in particular, CNs had to negotiate difficult travel conditions and to avoid the spread of the virus they were limited to two visits per day. According to CN Fiona, social isolation had been a big issue for clients, and she had been offering ‘mild psychological support’ such as cognitive behavioural therapy (CBT) online, although the main focus had been digital assistance for people with limited skills. In her view, it was frequently connecting people to family that made the most difference to clients’ lives:

I’ve got one lady who self-referred in for digital support who’d never had, absolutely no digital skills whatsoever, in her eighties, who would normally travel to see family in Australia . . . but obviously can’t, and it was then getting her device set up and teaching her how to use that. Now she Facetimes her family regularly, actually she Facetimes me on a Saturday just for a wee chat . . . It makes such a difference. (Fiona, CN)

### Isolation and lone working

In both rural and urban areas, the role of combatting loneliness and isolation – especially given social distancing – continued to be one of the pivotal aspects of the SPC role. Many examples were given of how social prescribers connected people, for example, SPCs in both the Western Isles and Glasgow referred people to an online befriending service:

One of the problems that Covid’s thrown up for social prescribing work is to get people who might otherwise be isolated to connect physically with other people . . . and that element of physical connection of people being in the same room for, at the moment, clearly that’s problematic – so a big thing that people want is befriending services. (Jerry, mPower)

Paradoxically, the enforced shift to online service provision and activities had benefitted clients previously unwilling or unable to attend physical, in-person sessions. Link worker Ray spoke of one lady who ‘doesn’t engage with anything in normal times because she doesn’t have the confidence . . . but I’ve linked her in with some online [yoga/Tai chi] sessions . . . and she’s really keen on that’. For this reason, it seemed likely that some services would continue online even post-pandemic. Thus, while the original concept of social prescribing was predicated on in-person relationships, social distancing measures and new technology platforms seem set to alter the landscape social prescribing for the foreseeable future.

Some of the link workers had not found home working easy: their work demanded a level of attention, confidentiality and privacy which could be hard to maintain on a shared household: ‘It’s making sure that I have my door closed and being aware of what my flat mates are doing as well . . . ’s just hard’. In terms of support for their home and online work, link workers had been offered extra training in such things as IT skills and data protection, although some of them felt that they needed more training ‘for issues that we’ve been dealing with since Covid started’. To mitigate against isolation, SPCs in all schemes had regular online meetings with line managers and colleagues. Managers from both the Community Links Worker and SPRING programmes thought that the pandemic had brought the importance of staff wellbeing and peer support into greater focus. In manager Lynne’s words:

We talk a lot about the patient experience and patient wellbeing, but we need to keep in mind that the links workers are humans as well. And they have, they’ll have family issues that, they’ll be touched by Covid, they’ll have childcare issues . . . and we need to be mindful of that and be supportive. Because I think sometimes there can be too high an expectation on a Link Worker.

### Diminished resources

In terms of the third sector, mPower manager Jerry remarked on the lower status historically afforded to the community sector, with a prioritizing of those within the NHS: ‘I’ve found that the way that the NHS approached [community services] was clunky, and very, very poor in terms of how it could, how it, how it connected and related with small community groups’.

Recent closure of local organizations before and since COVID had reduced the range of organizations to which SPCs could now refer their clients:

In Glasgow the council have pulled funding from a lot of organizations. So there was a women’s shelter for example, there was a local service that provided digital inclusion in the Govan area . . . There was a community cafe that a lot of my colleagues linked in with that lost funding . . . there’s one service I’ve referred a lot of people . . . they basically just lost funding to provide, just to send food parcels to people who need them around Glasgow. (Ranesh, CLP/link worker)

Some services had been adapting their services to online delivery, while new schemes such as the NHS *Compassionate Distress Service* had been set up directly in response to the pandemic. Optimism was expressed about the pace in which local communities had stepped in to fill voids and meet new needs. One Arts and Music programme – which before COVID had focused on employability – had set up a helpline offering emotional and practical support to local people. Nevertheless, providing one-to-one support over the phone was not ideal, and link worker manager Lynne knew of many clients waiting on services such as counselling to return to face-to-face delivery, which she predicted was likely to result in a backlog: ‘I see that as being a risk as we move forward; [these] services are going to be overwhelmed’.

### Future challenges

As for the future, there was recognition that many issues that social prescribing had sought to mitigate against, such as stress, social isolation and mental health issues, were likely to have increased, both as a result of the measures put in place to restrict the spread of the coronavirus and the subsequent strain on individuals and services, with those already disadvantaged most impacted. There was a general sense that social prescribing in its different forms would become more relevant in the coming months and years:

It [the pandemic is and will] put a massive strain on resources because the people aren’t allowed to go out that they’re, or not, aren’t working, there’s going to be increasing issues around mental health and finances and domestic abuse and alcoholism and so the need for a link worker helping people to access support is just going to be heightened. (Alison, researcher)

At the same time those employed as link workers or other SPCs still lack a defined professional status and with no obvious path to promotion and with organizational, management and funding structures shifting, it did not provide much job status or security for individual practitioners:

It’s a little bit unsettling, that’s the truth . . . when you start bringing organizations in who are tending for contracts and there’s a worry about your terms and conditions of contract, if they’re going to change your pay and all that type of thing, it’s unsettling. (Ray, CLP/ links worker)

## Discussion

Our findings bear out the diversity of the social prescribing landscape in Scotland and the complexity of cases which constitute the routine workload for the social prescriber. Within this overarching theme of system complexity we identified three subthemes: variations in referral criteria, variations in background and training and points of concordance. Previous studies have borne out the intensive case management of the link workers in Glasgow.^36^ The pandemic crisis appeared to have brought them more challenging and urgent referrals, such as related to mental health crises, financial debt and domestic violence. Those working for organizations outside GP practices (i.e. social prescribing advisors and CNs) were deemed less likely or equipped to deal with the ‘high-end’ cases such as suicide risk. Nevertheless, this study found many overlaps between different schemes in terms of their client base. In particular, all SPCs followed a person-centred approach, which as a tenet of social prescribing has been borne out in other studies.^1,5,21^

In the wake of the COVID-19 pandemic, this study highlights a number of challenges and shifts in focus for social prescribing schemes. These were discussed under the subthemes of remote working, changing demands, isolation and lone working, diminished resources and future challenges. Across all schemes, the use of multiple digital technologies had been essential to the delivery of social prescribing and, for practical and operational reasons, this situation seems likely to remain. This change of emphasis represents a real culture shift for social prescribing and one that impacts on clients with limited digital access or literacy. While more universally accessible, consultations over the telephone posed limitations in terms of confidentiality. Communicating with people for whom English was not a first language was flagged up as particularly unsatisfactory on the phone, suggesting that this cohort was likely to especially disadvantaged by social distancing measures. In the absence of an in-person interpreter, the availability of a telephone interpreting service had been essential, but as a method of communication, it had proved awkward and impersonal for all parties concerned.

A significant consequence of COVID-19 had been increased and changing demands on mainstream medical services. Our findings confirmed that, with clinical issues assuming so much GP attention, little time was left to focus on patients’ psychosocial issues, while some services, such as mental health, have been overwhelmed. Faced as professionals and their service users were with backlogs, some social prescribers had been taking on counselling and advocacy roles for the most serious cases. With many people stuck at home in difficult circumstances and out of work, SPCs had also been active in dealing with socioeconomic issues such as delivering medicines and attempting to tackle food poverty.

A general concern among stakeholders was the closure of important third sector services in local areas. This issue predates the pandemic and has been discussed in previous studies of social prescribing^11,22,36^ but appeared to have escalated by the close of this study. The folding of local services for youth is particularly concerning at a time when young people are largely deprived of social contact and usual routines and many young people are struggling with their mental health.^41^ Domestic abuse victim services in the United Kingdom have also seen a general increase in demand to helplines.^42^ While it is not part of the SPC remit to be duplicating such services, from our conversations with GPs and link workers, some appeared to be doing so where they could. These findings are in line with previous studies of the Links Worker Programme.^34^

Finally, this study brought to light issues around the work practices, employment and management of SPCs themselves. Concerns were expressed about the uncertainties over future funding, changes in management, lack of professional status and inadequate training, while the broad nature of the SPC role leaves it open to exploitation. Studies of previous pandemics suggest that health and social care workers have an increased risk of adverse mental health outcomes such post-traumatic stress disorder and depression.^43^ A study of lower paid health and social care staff in the Glasgow area indicated that these workers have higher rates of these conditions than people working in different sectors.^44^ SPCs are not strictly classified as working in health and social care; however they clients frequently present with problems such as depression, anxiety, bereavement, financial difficulties and may be “at the end of their tether”^45^, p.1 they largely work alone and now mostly at home; they lack a proper professional status, and some have insecure work contracts. All of these factors place an added strain on these workers which could be exacerbated by the more complex caseloads they receive in the wake of the pandemic.

## Conclusion

This study set out to examine the ways in which conditions created by the pandemic have reshaped and repurposed social prescribing, and how have they impacted on the role and responsibilities of the SPCs in urban and rural schemes. Our results confirm that significant challenges and shifts in focus in social prescribing have taken place in response to the pandemic. Digital technology has assumed a central role in social prescribing, shifting away from its traditional in-person focus. This study also brings to light the patchiness of the present social prescribing landscape, the eclectic and challenging nature of the SPC role and the additional pressures placed on practitioners now engaged in supporting heavily overburdened statutory services and a third sector under financial strain.

### Strengths and limitations

Limitations of this study including the failure to speak with patients (due to COVID-19 restrictions) and the relatively small sample of stakeholders and schemes contacted and interviewed. Strengths include the timeliness of the study and its unique perspective on the concept of social prescribing. We have been able to consider social prescribing in diverse settings within one country by speaking with various stakeholders of three different types of social prescribing schemes. This gave us a bird’s-eye view of social prescribing in Scotland, and from this, we could develop a high-level understanding of its benefits and challenges. Furthermore, by interviewing participants at the start and 11 months into the COVID-19 pandemic and lockdown, we were able to record how schemes adapted to the ongoing challenges.

### Recommendations

This study suggests the need for greater coordination and communication between the stakeholders involved in different social prescribing schemes. If the pandemic has taught us anything, it is the urgency that all health and social services are able to work together to tackle crises and that staff need to have the necessary support and training to do so. We consider that future research should focus on three areas of concern: the resilience and adaptability of social prescribing to health and social crises, a professionalization of the SPC role, and the inclusion of ethnic minorities in future social prescribing policy and planning arrangements.
